# Evaluation of completeness, consistency and non-duplication of leprosy notification data on the Notifiable Health Conditions Information System, João Pessoa, Paraíba, Brazil: a descriptive study, 2001-2019

**DOI:** 10.1590/S2237-96222023000200008

**Published:** 2023-05-08

**Authors:** Micheline da Silveira Mendes, André Luiz Sá de Oliveira, Haiana Charifker Schindler

**Affiliations:** 1 Fundação Instituto Oswaldo Cruz, Departamento de Saúde Coletiva, Recife, PE, Brazil; 2 Fundação Instituto Oswaldo Cruz, Núcleo de Estatística e Geoprocessamento, Recife, PE, Brazil; 3 Fundação Instituto Oswaldo Cruz, Departamento de Imunologia, Recife, PE, Brazil

**Keywords:** Leprosy, Health Information Systems, Public Health Surveillance, Epidemiology, Descriptive, Epidemiological Monitoring, Lepra, Vigilancia en Salud Pública, Sistemas de Información en Salud, Epidemiología Descriptiva, Monitoreo Epidemiológico, Hanseníase, Sistemas de Informação em Saúde, Vigilância em Saúde Pública, Epidemiologia Descritiva, Monitoramento Epidemiológico

## Abstract

**Objective:**

to analyze the completeness, consistency and non-duplication of leprosy notification data in João Pessoa, Paraíba, Brazil, 2001-2019.

**Methods:**

this was a descriptive study, conducted with data from the Notifiable Health Conditions Information System, which checked for “duplication” (acceptable: < 5%), “completeness” (excellent = incompleteness ≤ 5%) and “consistency” (excellent: ≥ 90.0%), based on the proportion of complete and consistent fields.

**Results:**

the sample consisted of 2,410 notifications. Duplication was acceptable (0.3%). The completeness of the “bacilloscopy”, “affected nerves”, “examined contacts” and “reactive episode” fields was very poor (more than 50% incomplete). Consistency between the “operational classification” and “initial treatment regimen” fields was excellent (99.6%), while consistency between “operational classification” and “clinical form” was low (50.7%).

**Conclusion:**

although duplication was acceptable, poor completeness of diagnosis and follow-up fields hinders epidemiological analysis, recognition of the status of the disease and adoption of measures to control it.

## Resumo

**Objetivo:**

analisar a completitude, consistência e não duplicidade dos dados da notificação da hanseníase em João Pessoa, Paraíba, Brasil, 2001-2019.

**Métodos:**

estudo descritivo, com dados do Sistema de Informação de Agravos de Notificação; verificou-se a duplicidade (aceitável quando < 5%), completitude (grau excelente = incompletitude ≤ 5%) e consistência (excelente quando ≥ 90,0%) dos dados, utilizando-se a proporção de campos preenchidos e coerentes.

**Resultados:**

a amostra de 2.410 notificações apresentou duplicidade aceitável (0,3%); a completitude dos campos “baciloscopia”, “n^o^ de nervos afetados”, “contatos examinados” e “episódio reacional” foi muito ruim (mais de 50% incompletos); e a consistência entre os campos “classificação operacional” e “esquema terapêutico inicial”, excelente (99,6%), enquanto entre “classificação operacional” e “forma clínica” foi baixa (50,7%).

**Conclusão:**

embora a duplicidade observada fosse aceitável, a completitude de campos sobre diagnóstico e acompanhamento foi ruim, dificultando a análise epidemiológica, o reconhecimento da situação do agravo e a adoção de medidas de controle da doença.

## Resumen

**Objetivo:**

analizar la completitud, consistencia y no duplicidad de los datos de notificación de lepra en João Pessoa, Paraíba, 2001 - 2019.

**Métodos:**

estudio descriptivo, realizado con datos del Sistema de Información de Enfermedades de Declaración Obligatoria, que verificó “duplicidad” (aceptable: < 5%). “completitud” (grado excelente: incompletitud ≤ 5%) y “consistencia” (excelente: ≥ 90,0%), utilizando la proporción de campos completos y coherentes.

**Resultados:**

se obtuvo una muestra de 2.410 notificaciones. La duplicidad se consideró aceptable (0,3%). La completitud de los campos “baciloscopía”, “nervios afectados”, “contactos examinados” y “episodio reaccional” fue muy pobre (más del 50% incompleta). La consistencia entre los campos “clasificación operativa” y “régimen terapéutico inicial” fue excelente (99,6%), mientras que “clasificación operativa” y “forma clínica” fue baja (50,7%).

**Conclusión:**

aunque la duplicidad es aceptable, la completitud de los campos de diagnóstico y seguimiento fue deficiente, lo que dificulta el análisis epidemiológico, el reconocimiento de la situación de la enfermedad y la adopción de medidas de control de la enfermedad.

**Table:** 

Study contributions	
**Main results**	In the sample of 2,410 cases the level of duplicaction was considered acceptable. However, the completeness of fields related to case follow-up was poor or very poor.
**Implications for services**	The low completeness of the data affects the epidemiological aspect and the planning of actions aimed at eliminating leprosy. This fact may, for example, compromise the acquisition of orthoses, necessary for the physical independence of people affected by the disease.
**Perspectives**	Investments in actions to raise awareness and qualify health professionals and epidemiological surveillance, to produce consistent data, with continuous monitoring, which contribute to demonstrating the epidemiological situation of leprosy.

## INTRODUCTION

Leprosy is an infectious disease, known for its magnitude and transcendence, caused by the *Mycobacterium leprae* bacterium, or Hansen’s bacillus, which affects the skin and peripheral nerves, and can lead to physical disabilities and deformities.^1^

In 1988, the World Health Organization (WHO) recommended that leprosy be classified based on skin lesions, for operational purposes. Since then, individuals with up to five skin lesions are classified as paucibacillary cases, while those with more than five skin lesions are classified as multibacillary cases.^2^

Leprosy is part of the group of neglected tropical diseases that are more endemic in regions where the population faces unfavorable living conditions, especially in underdeveloped countries.^1^

In this context, Brazil, India and Indonesia accounted for 74% of new leprosy cases worldwide in 2020.^3^ In that year, Brazil notified 17,979 new cases, representing a detection rate of 8.5 per 100,000 inhabitants, this being an indicator of average endemicity according to the criteria adopted by the Brazilian Ministry of Health.^4^ Specifically, the state of Paraíba and its capital, João Pessoa, also had average endemicity, with detection rates of 9.9 per 100,000 inhab. and 8.9 per 100,000 inhab. respectively.^5^

Leprosy is on the national list of compulsorily notifiable health conditions. Notifications are stored and managed on the Notifiable Health Conditions Information System (SINAN), which also enables recording of household contacts examined and case follow-up up until case closure. The SINAN data are used to calculate indicators that assist with gaining knowledge about the epidemiological behavior of the disease and with operationalization of care provided to infected people.^6^ ^),(^ ^7^

According to the United States Centers for Disease Control and Prevention (US/CDC) Guidelines for Evaluating Disease Surveillance Systems, evaluation of public health surveillance systems should seek to achieve efficient use of time and resources. Data validity and integrity are analyzed based on the following evaluation attributes: completeness, consistency and non-duplication.^8^

Analyzing notification form data is fundamental for epidemiological monitoring. This analysis takes place by identifying duplicated cases, form field completeness and consistency between the data relating to different notification variables.^8^ ^),(^ ^9^ ^)^ Poor filling out of the notification form, with incomplete or inconsistent data, can lead to unreliable analyses and the respective results thereof.

Completeness is understood to be a good characteristic of records, taking into account the extent to which the variables are filled out, estimated based on the proportion of forms with correctly filled out fields/variables.^10^ ^),(^ ^11^ Verification of the proportion of fields with missing information - recorded in the “unknown” category or simply not filled out (blank field) - is a way of measuring “completeness”, allowing the completeness and legitimacy of the records held on the system to be evaluated.^12^ ^)^ Implementing a rigorous evaluation routine helps to improve data quality and reliability, contributing to the control of diseases or health problems.^7^ ^),(^ ^8^

In the case of leprosy, there are few publications focusing on the evaluation of the notified leprosy data records held on the SINAN, despite its undeniable importance for epidemiological surveillance actions. The present study aimed to analyze duplication, completeness and consistency of leprosy notification records in the municipality of João Pessoa, state of Paraíba, Brazil, from 2001 to 2019.

## METHODS

This was a descriptive time series study, with secondary data obtained from notifications of leprosy cases held on the SINAN, in João Pessoa, between January 1, 2001 and December 31, 2019.

João Pessoa, capital of the state of Paraíba, is located in the Northeast region of Brazil. In 2021, its population was estimated at 825,796 inhabitants, with an urbanization rate of 99.6% and a municipal human development index considered high: 0.763.^13^ The municipal public health care service in João Pessoa carries out diagnosis and treatment of leprosy in its primary health care facilities, comprising 16 primary health care centers and 201 family health teams, as well as the Hospital Universitário Lauro Wanderley outpatient service, and an outpatient clinic specialized in the leprosy at the Hospital de Doenças Infectocontagiosas Dr. Clementino Fraga, which is a tertiary care referral service for the state of Paraíba.^14^

Notification forms of leprosy cases monitored in primary care are input at the headquarters of the Municipal Health Department, while notification forms from the hospitals are input at the local epidemiological surveillance services and sent via internet in weekly consolidated electronic files, all of which go to comprise the Municipal Health Department SINAN database. Once this flow is concluded, the data can be monitored and analyzed, sent to the state database, finally resulting in the production and availability of total information which can be broken down by municipality.

This study included all cases of leprosy reported on the SINAN for people resident in João Pessoa between 2001 and 2019. We defined 2001 as the start of the study period because it is the first year with data available on the municipal database. 2019 was defined as the last year of the study period because it was the last year with closed cases at the time when we accessed the SINAN database (April 2021) at the João Pessoa Municipal Health Department.

The variables selected for the study were extracted from fields classified as mandatory (if data is missing for these fields it is impossible to input the form on the system), essential (not mandatory, although necessary for calculating epidemiological or operational indicators) or other (complementary), according to SINAN criteria.^15^

For the purpose of analyzing completeness, the variables were divided into blocks according to the situation of case diagnosis and follow-up, as shown in Box 1.

**Box 1:** Variables selected for analysis of the completeness and consistency of leprosy notification records, João Pessoa, Paraíba, 2001-2019

Block	Variable name	Criterion
Diagnosis	Notifying health service	Mandatory
	Race/skin color	Essential
	Schooling	Essential
	Occupation	Complementary
	Operational classification	Mandatory
	Clinical form	Complementary
	Number of skin lesions	Complementary
	Initial treatment regimen	Mandatory
	Number of affected nerves	Complementary
	Assessment of physical disability grade at diagnosis	Essential
	Bacilloscopy	Complementary
	Number of recorded contacts	Essential
Follow-up	Follow-up health service	Mandatory
	Number of supervised doses received	Essential
	Reactive episode during treatment	Essential
	Number of contacts examined	Essential

Variables were considered to be incomplete when the field was not filled out (blank) or filled out as “unknown”. In the case of the “physical disability grade at diagnosis” and “bacilloscopy” variables, fields filled out with the options “not assessed” and “not performed”, respectively, were taken to be incomplete.

The adequacy of the records was verified according to the indicators for duplication, completeness and consistency proposed by Romero & Cunha^16^ and Abath et al.^17^ Duplicates were identified by analyzing the records organized by date of notification, comparing the names of the individual-case and their mother, the date of birth of the case and the date they started treatment. Based on these comparisons, cases that showed divergence were submitted to a manual review of the respective notification forms, so as to prove whether or not they were duplicated. Duplicate cases were excluded from the study, as per the criteria defined by Abath et al., whereby duplication below 5.0% was considered acceptable.^17^

Assessment of completeness was performed for each variable selected. After dividing the number of forms on which there was no information for the variable by the total number of notification forms, the quotient was multiplied by 100. The completeness criteria were the same as those proposed by Romero & Cunha (2006), taking the proportion of in-completeness^16^ (Box 2).

**Box 2:** Analysis of completeness, according to percentage incompleteness of the study reference

Degree of completeness	Proportion (%) of incompleteness
Excellent	< 5
Good	5-10
Regular	11-20
Poor	21-50
Very poor	> 50

In order to analyze consistency, that is, the level of coherence between one variable and another (absence of conflict between them), the records were aligned in four correlation scenarios: “operational classification” and “initial treatment regimen”; “operational classification” and “clinical form”; “operational classification” and “No. of skin lesions”; and “initial treatment regimen” and “clinical form”. The proportion of inconsistent records was calculated after excluding cases for which these fields were either not filled out or were filled out as “not classified”. In the pairing between “operational classification” and “No. of cutaneous lesions”, cases defined as paucibacillary but with more than five skin lesions were considered inconsistent. The classification proposed by Abath et al.^17^ was adopted in order to assess consistency: excellent, when consistency percentages are equal to or greater than 90.0%; regular, between 70.0% and 89.0%; and low, when below 70.0%.

The data obtained, processed and analyzed using Microsoft Excel software were made available in graphs and simple frequency and proportion distribution tables. In the case of completeness analysis, case diagnosis and follow-up variables were presented according to health facility.

The study project was conducted in accordance with the recommendations of National Health Council Resolution No. 466, dated December 12, 2012, and was approved by the Instituto Aggeu Magalhães/Fundação Instituto Oswaldo Cruz (Fiocruz) Research Ethics Committee - File No. 4.573.230.

## RESULTS

A total of 2,418 leprosy case notifications were recorded on the SINAN in the municipality of João Pessoa between 2001 and 2019. Of this total, 8 (0.3%) duplicate notifications were excluded, resulting in 2,410 notification forms: 1,717 cases diagnosed at the state referral service, 579 at municipal primary care services and 114 at the Hospital Universitário Lauro Wanderley.

**Figure 1:**
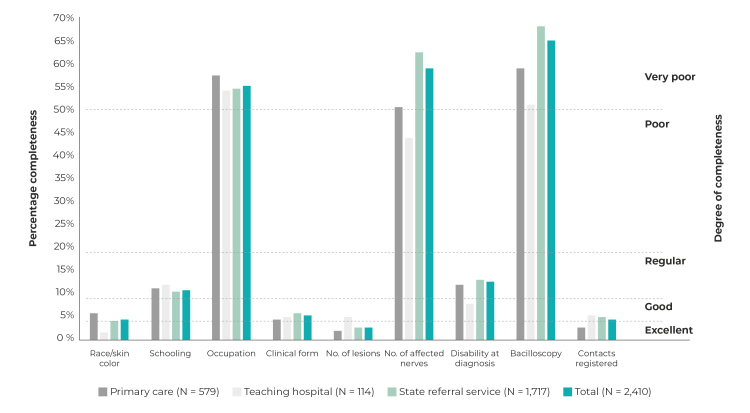
Percentage incompleteness of essential fields on the leprosy case notification form on the Notifiable Health Conditions Information System, and degree of completeness, by notifying health service, João Pessoa, Paraíba, 2001-2019

Completeness was found to be good and excellent for the “race/skin color”, “clinical form”, “No. of skin lesions” and “No. of contacts registered” fields. Completeness of the “schooling” and “disability grade at diagnosis” variables was regular (10%-19% of fields incomplete), while completeness of “occupation”, “No. of nerves affected” and “bacilloscopy” was very poor (over 50% of fields incomplete), for all types of health services (Figure 1).

**Figure 2:**
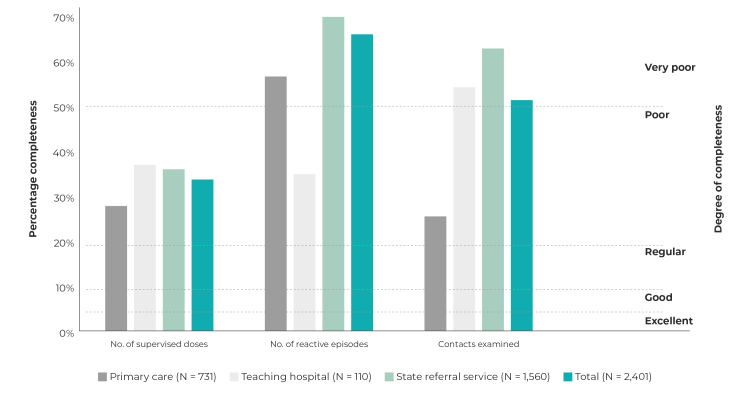
Percentage of incomplete fields related to leprosy case follow-up on the Notifiable Health Conditions Information System, and degree of completeness attributed, by follow-up health service, João Pessoa, Paraíba, 2001-2019

Figure 2 shows the percentage completeness of variables related to case follow-up. Completeness of the “No. of supervised doses” variable was poor (from 20% to 50% of fields not completed), completeness of “reactive episode” and “No. of contacts examined” was classified as very poor (above 50% of fields without information), whereby the percentage of fields not completed was more significant for the state referral service: 72.9% “reactive episode” and 63.3% “No. of contacts examined” fields not filled out.

When considering the consistency of the variables, forms without information in the evaluated fields were excluded, thus impacting the total number of forms for each pair of fields analyzed. As such, the following were excluded: 8 (0.3%) forms when analyzing the “operational classification” and “initial treatment regimen” fields; 77 (3.2%) forms for “operational classification” and “No. of skin lesions”; and 142 (5.9%) forms for “operational classification” and “clinical form”, “clinical form” and “initial treatment regimen” (Table 1).

The consistency analysis considered coherence between the information recorded in the “operational classification” and “initial treatment regimen” fields, with an excellent result (equal to or above 90% consistency) in total. However, when analyzing consistency between the pairs of records in the “operational classification”, “clinical form”, “initial treatment regimen” and “No. of skin lesions” fields, low or regular consistency was found (Table 1).

**Table 1:** Percentage coherence between paired variables for leprosy case notifications held on the Notifiable Health Conditions Information System, and classification of total consistency, João Pessoa, Paraíba, 2001-2019

Variáveis	Total			Consistency classification
	N	Coherent forms	%	
Operational classification and initial treatment regimen	2,402	2,392	99.6	Excellent
Operational classification and clinical form	2,268	1,150	50.7	Low
Operational classification and number of skin lesions	2,333	1,952	83.7	Regular
Clinical form and initial treatment regimen	2,268	1,156	51.0	Low

## DISCUSSION

Considering the criteria evaluated in the present study, based on the records of leprosy cases held on the SINAN in the municipality of João Pessoa for the period between 2001 and 2019, we found that all follow-up fields (essential) evaluated, and three diagnosis fields (complementary), had a poor or very poor degree of completeness, regardless of the type of health facility. The results showed excellent consistency only when consistency between two mandatory variables was analyzed. However, the percentage of duplicate records was found to be acceptable. This finding indicates that duplicates were controlled; however, there are failures in filling out the fields and in monitoring the system that compromise the information produced through the SINAN, leading to errors in interpretation and, consequently, in the planning of actions in the face of this health condition.

The study’s limitations include those related to the use of secondary data from the data recording system. Secondary data can produce biases by underestimating the number of people affected, thus impacting the indicators. There is no way to quantify underreporting, although a reduction in this risk is to be expected, since the first level of grouping of data from all services in the municipality was used, in addition to medication packaged and sent by pharmaceutical care services following case notification, which has been done for the last decade. Another limitation of this work is related to the limited availability of studies on the completeness of leprosy data, making it difficult to make comparisons with data from other studies.

Some 71% of notifications containing diagnosis of leprosy cases, during the study period, were made by the state referral service; in disagreement with what the Ministry of Health advocates, that is, decentralization of leprosy control actions to Primary Health Care with emphasis on tracing, diagnosis, treatment, prevention, surveillance and combating social stigma associated with leprosy.^18^ In Brazil, the main strategy for decentralizing leprosy control actions consists of expanding the coverage of Family Health teams, expanding the population’s access to health services and thus making early diagnosis and timely treatment possible. However, the concentration of diagnosis in the referral service demonstrates that the challenges of decentralization go beyond increasing the coverage of Primary Care. A study on the epidemiology of leprosy and its association with the decentralization of disease control actions in Brazilian municipalities, between 2001 and 2015, points to the need for changes in the training and employment of health professionals, in health team work processes and in the priorities of the health service management’s political agenda, so that the decentralization of leprosy case tracing, diagnosis and follow-up activities becomes effective.^19^ Notwithstanding, a study conducted out in Teresina, Piauí, in 2012, found a different distribution, with a higher proportion of diagnoses in Primary Care.^20^

Primary Care is considered the main gateway to the health system and its capillarity is linked to advances in decentralized care, identification of suspected cases, diagnosis, follow-up and examination of contacts, while the referral service serves as a backup, for more complex diagnoses, investigation of drug intolerance, relapses, therapeutic resistance and prescription of replacement treatment regimens.^21^

The analysis of the records used in this study found only 8 (0.3%) duplicate records, this being a proportion considered acceptable, corroborating the results found in the state of Mato Grosso (99.5%) regarding leprosy in children under 15 years of age, between 2001 and 2013, ^(^ ^22^ as well as those of an evaluation of indicators for the state of Pernambuco between 2005 and 2014.^23^ Despite the proportion of duplicates found (0.3%) being acceptable, the João Pessoa municipal surveillance service needs have a routine for finding duplicates, since repeated notifications lead to overestimation of case prevalence, thereby impacting the results of studies, calculation of indicators and planning of actions.

With regard to our analysis of the filling out of the variables reported by the first notifying health facility, the degree of completeness was considered good or excellent for the “race/skin color”, “clinical form”, “No. of skin lesions” and “No. of contacts registered” variables. These results draw attention, since for the variables in question, despite their being essential for case characterization and active tracing of new cases, filling them out is not mandatory. The result we obtained for the “race/skin color” variable is similar to that of a national analysis conducted between 2016 and 2020, when missing information in this field accounted for just 3.4%;^24^ as well as being similar to the 3.7% found in Londrina, state of Paraná, from 2009 to 2016, with excellent completeness.^25^ Filling out this field contributes to analyses of social inequalities and risk of illness, and to building public policies that consider the needs of different ethnic-racial groups.

Based on the analysis of the filling out of the “No. of nerves affected” field, we found that the degree of completeness was poor or very poor, regardless of the type of health service. However, the “disability grade at diagnosis” had good completeness at the Hospital Universitário Lauro Wanderley, while it was regular at the state referral service and at the primary care facilities. Physical disability at diagnosis indicates operational aptitude for early diagnosis.

Completeness of the “bacilloscopy” field was very poor. It is neither a mandatory nor an essential field for filling out on the notification form. Its use is recommended when there is certainty that testing is available. Slit-skin smear bacilloscopy is an auxiliary examination for diagnosis and classification of the disease, in addition to assisting with defining leprosy relapse. This poor performance may be a consequence of centralizing sample collections and tests at a health service, as well as its non-mandatory nature. Similar findings were found in studies carried out in Bahia, between 2001-2014 and 2005-2015, and in Mato Grosso, between 2001 and 2013, with individuals under 15 years of age.^25^

As for case follow-up, the “No. of supervised doses” field was completed in less than 50% of the cases monitored, at all levels of health services. This finding is worrying, because monitoring the monthly dose is an opportunity to assess the health service user and reinforce guidelines on treatment and self-care. Monitoring monthly doses makes it possible to identify service users who fail to attend, and they should receive a home visit within 30 days, in order to avoid treatment abandonment.^21^

In general, recording of the “No. of contacts examined” during treatment scored a very poor degree of completeness. Completing this field correctly contributes to assessment of the capacity of health services in performing surveillance actions aimed at this group, as well as contributing to building the operational indicator that verifies the proportion of leprosy contacts examined, among those recorded.^23^ ^),(^ ^24^ A study carried out in 2014 in the municipality of Cacoal, state of Rondônia, in Northern Brazil, found low completeness for dermatological and neurological clinical evaluation of contacts, and reiterated the need for more evidence-based health education actions.^25^

Examination of contacts is the main active tracing activity for new cases, among people who live or have lived with a person affected by leprosy. Individuals identified as contacts of leprosy cases represent the group with the highest risk of contracting leprosy compared to the general population. Active tracing promotes early diagnosis and prevention of disabilities and permanent sequelae caused by the disease. Examining contacts is a fundamental strategy for surveillance and breaking the leprosy transmission chain.^25^

The consistency of the information system evaluated in this study, especially when pairing “clinical form” with “operational classification” or with “initial treatment regimen”, proved to be low. However, when “operational classification” was paired with “treatment regimen”, excellent consistency was found between both variables. The pairing of “operational classification” with “No. of skin lesions” revealed regular consistency, impacted by paucibacillary cases with more than six skin lesions. Similar findings were found for Teresina, capital of the state of Piauí, in 2012.^20^ As such, the importance of knowing the different clinical presentations of the disease and guaranteeing the recording of data was demonstrated, especially when classification mistakes occur.

In conclusion, weaknesses were identified in the records of information with regard to case diagnosis, as well as in case follow-up, this being essential in order to avoid treatment abandonment, carry out surveillance of contacts and achieve a favorable outcome. Monitoring and systematic evaluation of the completeness, consistency and non-duplication of input to the SINAN is confirmed as a necessary activity for epidemiological surveillance of leprosy in the city of João Pessoa.
